# A Universal Pharmacokinetic Model for Dexmedetomidine in Children and Adults

**DOI:** 10.3390/jcm9113480

**Published:** 2020-10-28

**Authors:** James D. Morse, L. Ignacio Cortinez, Brian J. Anderson

**Affiliations:** 1 Department of Pharmacology & Clinical Pharmacology, University of Auckland, Park Road, 1023 Auckland, New Zealand; j.morse@auckland.ac.nz; 2 Department of Anesthesiology, School of Medicine, Pontificia Universidad Católica de Chile, Santiago de Chile 8331150, Chile; icortinez@gmail.com; 3 Department of Anaesthesiology, University of Auckland, Park Road, 1023 Auckland, New Zealand

**Keywords:** anaesthesia, intravenous, dexmedetomidine, children, obesity, total intravenous anaesthesia (TIVA), target-controlled infusion, pharmacokinetics, pharmacodynamics

## Abstract

A universal pharmacokinetic model was developed from pooled paediatric and adult data (40.6 postmenstrual weeks, 70.8 years, 3.1–152 kg). A three-compartment pharmacokinetic model with first-order elimination was superior to a two-compartment model to describe these pooled dexmedetomidine data. Population parameter estimates (population parameter variability%) were clearance (CL) 0.9 L/min/70 kg (36); intercompartmental clearances (Q2) 1.68 L/min/70 kg (63); Q3 0.62 L/min/70 kg (90); volume of distribution in the central compartment (V1) 25.2 L/70 kg (103.9); rapidly equilibrating peripheral compartment (V2) 34.4 L/70 kg (41.8); slow equilibrating peripheral compartment (V3) 65.4 L/70 kg (62). Obesity was best described by fat-free mass for clearances and normal fat mass for volumes with a factor for fat mass (FfatV) of 0.293. Models describing dexmedetomidine pharmacokinetics in adults can be applied to children by accounting for size (allometry) and age (maturation). This universal dexmedetomidine model is applicable to a broad range of ages and weights: neonates through to obese adults. Lean body weight is a better size descriptor for dexmedetomidine clearance than total body weight. This parameter set could be programmed into target-controlled infusion pumps for use in a broad population.

## 1. Introduction

Total intravenous anaesthesia (TIVA) has been widely practiced in adult anaesthesia since the introduction of propofol into routine clinical practice in 1982. Use of TIVA has expanded from drug delivery rates (mass infusion rates) that require frequent manual changes by the clinician to maintain the target concentration. Target-controlled infusion (TCI or “smart”) pumps produce a user-defined drug concentration in either the plasma (Cp) or at the effect site of action (Ce) [1].

Drug delivery is determined by known pharmacokinetic (PK) parameter sets (e.g., population clearance (CL) and volume (V) estimates) that are programmed into these target-controlled pumps. Adult parameter sets are published for propofol [2,3], remifentanil [4], sufentanil [5], alfentanil [6], dexmedetomidine [7], and ketamine [8]. Parameter sets for children have also been published for propofol [9,10], remifentanil [11], and dexmedetomidine [12]. However, propofol currently remains the only drug available for children in many TCI pumps. Due to this scarcity of paediatric PK models in commercially available TCI pumps, adult PK parameters (e.g., those described by Minto et al. for remifentanil [4]) continue to be used in TCI pumps for children, despite both V and CL (expressed as mL/min^/^kg) decreasing with increasing age [11,13].

PK models that encompass a wide range of patient groups (e.g., children, adults and the obese) have been reported for propofol [14] and remifentanil [15]. These newer models use allometric theory, maturation concepts and consider fat mass in their description as “universal” models. One of the greatest advantages of these models is simplification of the administration of TCI by allowing a single model to be able to administer the drug in widely different groups of patients. Dexmedetomidine PK have been described in divergent groups of patients and conditions (e.g., healthy volunteers [7], children [12], normal weight and obese adult patients [16,17]). No universal model has been derived for its administration in TCI.

Dexmedetomidine PK have also been described in children using allometric scaling [18,19]. If an adult dexmedetomidine PK model that was scaled for size using allometry was applicable to children older than 1 year, then it should be possible to construct a universal dexmedetomidine PK model using pooled data applicable to children and adults [20]. This model should also be applicable to obese adults by considering the nonlinear relationship between fat mass and dexmedetomidine clearance [21].

## 2. Methods

### 2.1. Data Sources

Data for development of the universal model were sought from five previously published studies of dexmedetomidine PK.

1. Hannivoort Model: Hannivoort and colleagues [7] recruited 18 (9 male and 9 female) individuals 18–72 years old with BMI scores between 18 and 30 kg/m^2^. Dexmedetomidine was delivered using the Dyck model [22] targeting concentrations of 1, 2, 3, 4, 6, and 8 ng/mL after an initial infusion of 6 µg/kg/h for 10 s. Each step was maintained for 30 min. Blood samples for dexmedetomidine assay were obtained at 2 minutes after the initial drug infusion, before each increase in target concentration and at 2, 5, 10, 20, 60, and 120 min after the drug infusion stopped. A large local database [23] was used to sample 18 individuals representative of the demographics in the Hannivoort population. Simulated predicted concentrations in these 18 individuals given 2 mcg/kg loading dose over 10 min followed by infusion 1 mcg/kg for 2 h were used to develop the universal model.
2. Potts Model: Potts and colleagues [12] recruited 45 children (22 males and 23 females) after cardiac surgery. Dexmedetomidine was administered (1–4 µg/kg) over 10 min. Three to four blood samples were obtained in the first 30 min after infusion. Samples were obtained at 1–2, 3–4, and 6–10 h thereafter. These data were pooled with two other PK studies (*n* = 34) of dexmedetomidine [24,25]. These studies are summarised in Supplementary Materials Table S1.
3. Cortinez Model: Cortinez and colleagues [16] recruited 20 obese (BMI >35 kg/m^2^) and 20 non-obese individuals (BMI 18.5–30 kg/m^2^, 18–60 years old), undergoing elective laparoscopic surgery. Dexmedetomidine 0.5 µg/kg was given to all participants for 10 min. Subsequently, participants were randomised to two infusion regimens: 0.25 or 0.5 µg/kg/h. Doses were based on total body weight (TBW). Blood samples were obtained at 2, 5, 10, 15, 20, 30, 45, 60, 90, and 120 min during dexmedetomidine infusion and at 0, 2, 5, 10, 20, 30, 60, 90, 120, 240, and 360 min after the infusion was stopped.
4. Rolle Model: This study enrolled 40 adults (age 18 to 60 years, weights 47 to 126 kg, BMI 18–49 kg/m^2^) scheduled for abdominal laparoscopic surgery [17]. Dexmedetomidine bolus of 0.5 mcg/kg over 10 min was followed by an infusion of 0.5 mcg/kg/h. Venous blood samples were drawn at 0, 5, 10, 20, 30, 45, 60 min after the start of dexmedetomidine administration and thereafter every 30 min during anaesthesia maintenance. Once dexmedetomidine infusion was stopped at the end of surgery, samples were drawn at the end of dexmedetomidine infusion, and then 5, 10, 20, 30, 60, 90, 120, 240, 360 min, with a last sample between 720 and 1200 min.
5. Talke Model: Talke and colleagues recruited 10 healthy individuals (21–36 years old and 52–89 kg) [26]. Dexmedetomidine 4 µg/mL was administered for 15 min to target a plasma concentration of 0.3 ng/ml. Blood samples were obtained at 1, 2, 3, 4, 5, 7.5, 10, and 15 min during drug infusion and 15, 30, and 60 min after the end of the infusion.

### 2.2. Hannivoort Model Performance in Children Older Than 1 Year

The adult PK model described by Hannivoort and colleagues [7] was used to simulate dexmedetomidine concentrations in the paediatric population at each time point where there was a concentration measurement in the data from Potts et al. [19] The predictive performance of this adult model was assessed using methods proposed by Sheiner and Beal [27]. The prediction error (pe) was calculated according to Equation (1).

(1) $$\mathrm{pe}\;=\;\left(C_{p}-C_{o}\right)$$

where C_0_ is the observed concentration and C_p_ is the concentration predicted by the adult model. Precision was evaluated using the root mean squared prediction error (rmse) (Equation (2)).

(2) $$\mathrm{mse}\;=\;\frac{1}{N}\overset{N}{\underset{i=1}{\sum }}{\mathrm{pe}}_{i}{}^{2};\;\mathrm{rmse}=\;\sqrt{\mathrm{mse}}$$

The mean prediction error was used to calculate bias (Equation (3)).

(3) $$\mathrm{me}\;=\;\frac{1}{N}\overset{N}{\underset{i=1}{\sum }}{\mathrm{pe}}_{i}$$

### 2.3. Pooled Data Analysis

These five datasets (Hannivoort [7], Potts [19], Cortinez [28], Rolle [17], Talke [26]) were pooled to investigate the effects of size, age, and fat mass on descriptive PK and to construct a universal model applicable to infants, children, and adults including the obese.

#### 2.3.1. Pharmacokinetic Analyses

Two and three-compartment PK models with first order elimination were used to describe dexmedetomidine PK. These models were parameterised in terms of CL, V, and intercompartmental clearance (Q). Allometric theory described the relationship between size, structure, and function and was used to quantify size-related changes in PK parameters [29]. PK parameters (e.g., CL, Q, V) were standardised to an adult measure of body size (size) with a standard weight of 70 kg using allometric scaling (Equation (4)) [30,31,32].

(4) $$\mathrm{Fsize}\;={\left(\frac{\mathrm{size}}{70}\right)}^{\mathrm{EXP}}$$

(5) $${\mathrm{CL}\;=\;\mathrm{CL}}_{\mathrm{STD}}\;\times \;\mathrm{Fsize}$$

where Fsize is a variable describing the fractional difference from a standard adult value and EXP is the allometric exponent, ¾ for functional processes such as clearance and 1 for volumes. Equation (5) shows how Fsize can be used to scale a standard value of CL (CL_STD_) to predict the value in a given individual.

Population parameter estimates were obtained using nonlinear mixed effects models (NONMEM 7.4 ICON Development Solutions, USA) with first-order conditional estimation and a convergence criterion set to 3 significant digits.

Population parameter variability (PPV) was accounted for using an exponential model for the random effect variables (η). This assumes a log-normal distribution and avoids parameter estimates falling below biologically plausible values. Variables were assumed to have a mean of zero and variance denoted by ω^2^ (Equation (6)).

(6) $$P_{i}{\;=\;P}_{\mathrm{TV}\;}e^{\eta i}$$

where P is the parameter (e.g., CL) for the *i*th individual, P_TV_ is the typical value for that parameter, and η is the random effects variable.

Residual unidentified variability (RUV) was modelled using both proportional and additive residual errors (Equation (7)). The between subject variability (η_RUV,i_) of the RUV was also estimated for both PK and PD data. The population mean parameters, between subject variance and residual variance, were estimated using the first-order conditional interaction estimate method using ADVAN13 TOL=9 of NONMEM. Convergence criterion was 3 significant digits.

(7) $${\mathrm{SD}}_{\mathrm{ij}}\;=\;\sqrt{\left({\left({\mathrm{Obs}}_{\mathrm{ij}}{.\theta }_{\mathrm{RUV}\_\mathrm{CV}}\right)}^{2}+\;{\left(\theta_{\mathrm{RUV}\_\mathrm{SD}}\right)}^{2}\right).}e^{{\eta \mathrm{PPV}}_{\mathrm{RUV}\;i}}$$

where Obs_ij_ is the dexmedetomidine plasma concentration in the *i*th individual at the *j*th time. Individual predictions of dexmedetomidine concentration were calculated using Equation (8) with the random effects (ε) fixed to 1.

(8) $${Y=\;\mathrm{Obs}}_{\mathrm{ij}}\;+\;{\mathrm{SD}}_{\mathrm{ij}\cdot \varepsilon }$$

#### 2.3.2. Covariate Analysis for Age and Size

The influence of body composition on the PK parameters CL and V were investigated using total body mass (kg), fat-free mass (FFM, kg), and normal fat mass (NFM, kg). Measurements of FFM were available in the Cortinez and Rolle data [16,17] and were determined using dual X-ray absorptiometry. FFM was not available for the pooled paediatric data. Predictive equations (Equations (9) and (10)) derived by Al-Sallami and colleagues [33]) were used to calculate FFM in this population.

(9) $$\mathrm{FFM}\left(\mathrm{males}\right)\;=\;\left[0.88\;+\;\left(\frac{\left(1-0.88\right)}{\left[1\;+{\left(\frac{\mathrm{Age}}{13.4}\right)}^{-12.7}\right]}\right)\right]\;\times \;\left[\frac{\left(9270\;\times \;\mathrm{Weight}\right)}{6680\;+\;\left(216\;\times \;\mathrm{BMI}\right)}\right]$$

(10) $$\mathrm{FFM}\left(\mathrm{females}\right)\;=\;\left[1.11\;+\;\left(\frac{\left(1-1.11\right)}{\left[1\;+{\left(\frac{\mathrm{Age}}{7.1}\right)}^{-1.1}\right]}\right)\right]\;\times \;\left[\frac{\left(9270\;\times \;\mathrm{Weight}\right)}{8780\;+\;\left(244\;\times \;\mathrm{BMI}\right)}\right]$$

For the adult data sourced from Talke [26], FFM was predicted using Equations (11) and (12). [34].

(11) $$\mathrm{FFM}\left(\mathrm{adult}\;\mathrm{males}\right)\;=\;\left(\frac{\left(9270\;\times \;\mathrm{Weight}\right)}{6680\;+\;\left(216\;\times \;\mathrm{BMI}\right)}\right)$$

(12) $$\mathrm{FFM}\left(\mathrm{adult}\;\mathrm{females}\right)\;=\;\left(\frac{\left(9270\;\times \;\mathrm{Weight}\right)}{8780\;+\;\left(244\;\times \;\mathrm{BMI}\right)}\right)$$

Normal fat mass (NFM) is a size descriptor based on allometric theory describing contributions from fat mass and FFM. NFM is FFM plus a component of fat mass, Equation (13), which can be described using the parameter *Ffat*; see [21].The effect of FFM on CL and V was assessed by fixing FFAT to zero (i.e., considering the effect of FFM alone).

(13) FAT = TBW − FFM

(14) NFM = FFM + Ffat × FAT

Allometric body mass can be determined using a standard value for NFM known as NFM_STD_. The standardised value for NFM can be defined using a FFM of 56.1 kg, expected for a male with a TBM of 70 kg and height of 1.76 m. Theory-based allometric scaling can be used to compare CL values for a child in terms of a standardised NFM value, most widely expressed for a 70 kg individual, with the allometric exponent of ¾. This is shown in Equation (15).

(15) $${\mathrm{CL}}_{\mathrm{Child}}{\;=\;\mathrm{CL}}_{\mathrm{STD}}\;\times \;{\left(\frac{{\mathrm{NFM}}_{\mathrm{Child}}}{{\mathrm{NFM}}_{\mathrm{STD}}}\right)}^{3/4}$$

The effects of size and body composition on drug PK can be predicted using NFM, allometric theory, and separation of body mass into its fat and fat-free components [35].

The maturation of dexmedetomidine CL was described using a maturation function (Equation (16))

(16) $$\mathrm{MF}\;=\;\frac{{\mathrm{PMA}}^{\mathrm{Hill}}}{{\mathrm{TM}}_{50}^{\mathrm{Hill}}{\;+\;\mathrm{PMA}}^{\mathrm{Hill}}}$$

where MF is the maturation factor, *PMA* is postmenstrual age in weeks, *TM_50_* is the maturation half-time, and the *Hill* exponent relates to the steepness of the maturation profile [31].

#### 2.3.3. Model Selection

The minimum value of the objective function (OBJ (−2log-likelihood (−2LL))) provided by NONMEM served as a guide during model building. Model selection was also based on parameter plausibility and prediction-corrected visual predictive check (PC-VPC) plots [36]. For two nested models, a decrease in the minimum value of the objective function (ΔOBJ) of 3.84 points for an added parameter was considered significant at the 0.05 level. Shrinkage considers the quality of the observed data. The term η-shrinkage refers to the between subject variability. When the observed data are informative, η-shrinkage approaches zero, and when the data are less informative, it approaches 1. Bootstrap methods provided a means to evaluate parameter uncertainty [37]. A total of 1000 bootstrap replications were used to estimate parameter means and confidence intervals. Results from the population models are presented as parameter estimates, together with their 95%CI. Between subject parameter variability is expressed as an apparent coefficient of variation obtained from the square root of the variance estimate (CV (%)).

### 2.4. Model Simulation

A dexmedetomidine plasma concentration > 0.6 mcg/L is estimated to produce adequate sedation in adult ICU patients [38]. A target of 1 mcg/L has been estimated for those adults and children out of the intensive care environment [39,40]. Since dexmedetomidine is commonly used at fixed infusion rates, we have simulated dexmedetomidine maintenance dose (mcg/kg/h) in a representative group of patients to achieve a target concentration of 1 mcg/L. Simulations were performed using typical values as estimates of volumes and clearances with the PKPD Tools software (freely available at http://pkpdtools.com/).

## 3. Results

**Figure 1:**
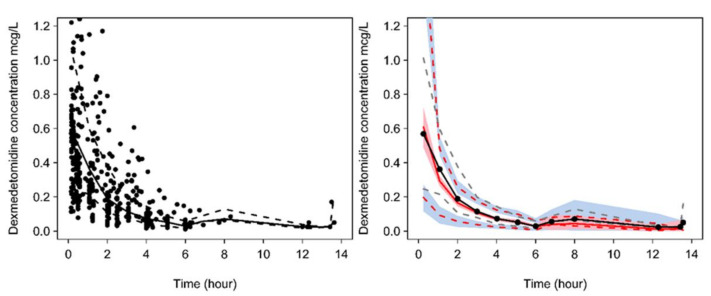
Prediction-corrected visual predictive check (PC-VPC) for dexmedetomidine pharmacokinetics using the model by Hannivoort [7] with observed dexmedetomidine plasma concentrations sought from Potts [19]. Plots show median (solid) and 90% intervals (dashed lines). The left-hand plot shows all prediction-corrected observed dexmedetomidine concentrations. Right-hand plot shows prediction-corrected percentiles (10%, 50%, and 90%) for observations (grey dashed lines) and predictions (red dashed lines) with 95% confidence intervals for prediction percentiles (median, pink shading; 5th and 95th blue shading).

The Hannivoort model [7], when used to predict dexmedetomidine concentrations in the paediatric cohort, had a precision of 22% and bias of 3.3%. The observed/predicted dexmedetomidine ratios for the Hannivoort model when used to predict concentrations in the population sourced from Potts are shown in Supplementary Materials Figure S1. An observed/predicted ratio of 1 would suggest that the model predictions are identical to observed concentrations. The PC-VPC shown in Figure 1 demonstrated that the Hannivoort three-compartment model [7] predicts concentrations observed by Potts et al. [19].

Parameter estimates for the pooled data analysis determining the “universal” model are shown in Table 1.

**Figure 2:**
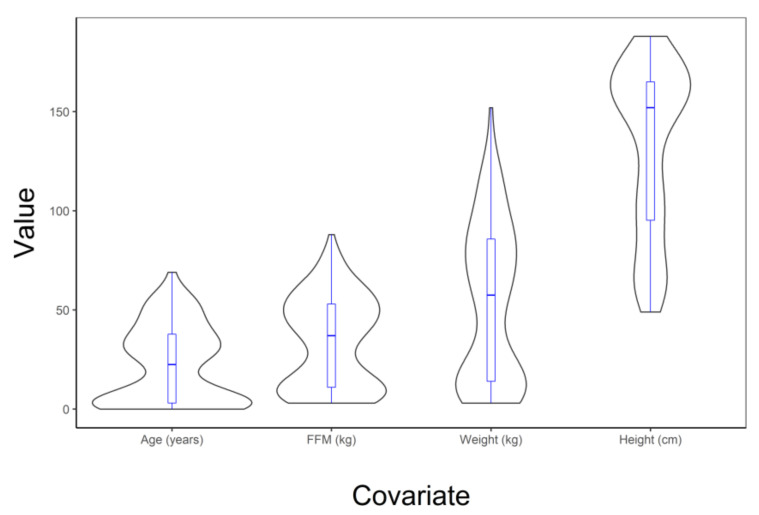
Violin plot showing the distribution of age (years), fat-free mass (kg), weight (kg), and height (cm) in the pooled dexmedetomidine data used to develop the universal PK model. A box and whisker plot overlays the violins in blue.

There were 202 individuals with 2145 dexmedetomidine concentrations that were amenable for modelling in the pooled dexmedetomidine PK analysis. Violin plots illustrate the distribution of covariates in this pooled population (Figure 2).

**Figure 3:**
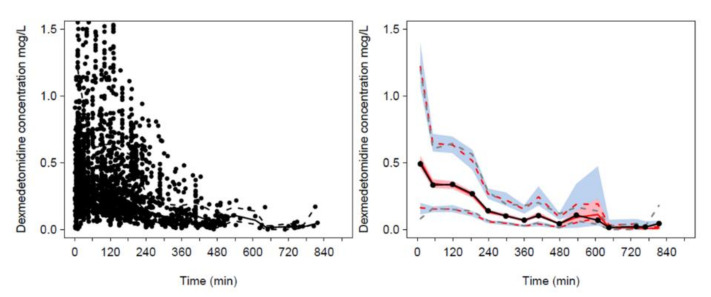
Prediction-corrected visual predictive check (PC-VPC) for the universal dexmedetomidine PK model. Model developed using pooled paediatric [19] and adult [16,26] dexmedetomidine plasma concentrations. Plots show median (solid) and 90% intervals (dashed lines). The left-hand plot shows all prediction-corrected observed dexmedetomidine concentrations. Right-hand plot shows prediction-corrected percentiles (10%, 50%, and 90%) for observations (grey dashed lines) and predictions (red dashed lines) with 95% confidence intervals for prediction percentiles (median, pink shading; 5th and 95th blue shading).

**Figure 4:**
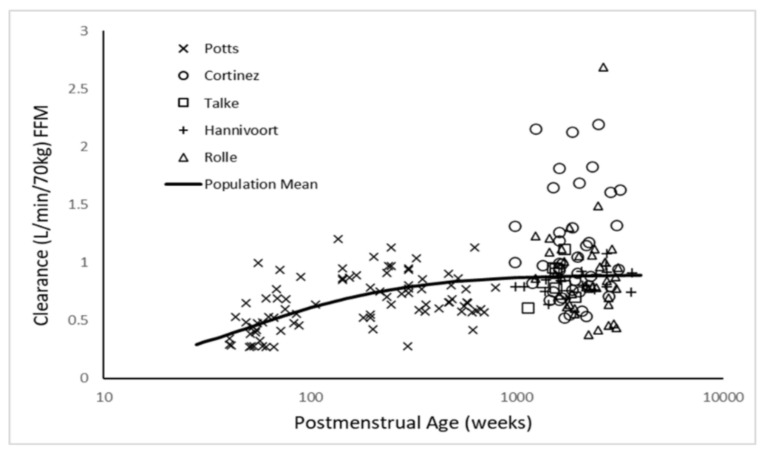
Maturation of dexmedetomidine clearance when scaled using fat-free mass, determined from pooled published data.

The three-compartment PK model proved superior to the two-compartment model for the pooled dexmedetomidine analysis (ΔOBJ 285.2). Use of allometric scaling was better than per kilogram scaling (ΔOBJ 25.331). Addition of maturation function for clearance further improved the model (ΔOBJ 20.058). The best model incorporated allometric scaling of CL and V with the changes in CL due to age accounted for with a maturation function. FFM proved to be a better PK size descriptor for clearance than TBW (ΔOBJ 40.9). Fat mass had an effect on volume (FfatV = 0.293). The PC-VPC for the universal dexmedetomidine model is shown in Figure 3. The maturation of dexmedetomidine CL is shown in Figure 4 when scaled using FFM and in Supplementary Figure S2 when scaled using TBW. The slope of this maturation function, defined by the Hill exponent, was initially estimated as 1.15 (95%CI 0.79, 1.90). Fixing this parameter to unity resulted in a minimal objective function increase (ΔOBJ 2.1) and no change in model performance.

The ratio of the between subject variability (BSV) predictable from covariates (BSVP) to the total population parameter variability obtained without covariate analysis (PPVt) gives an indication about how important covariate information is (Table 2). BSVR is the random BSV estimated on a parameter when covariate analysis is included. The ratio of 0.867 achieved for clearance in this current study indicates that 86.7% of the overall variability in clearance is predictable from covariate information; most is attributable to allometric scaling (83.8%). The use of FFM for size had minor impact on clearance between subject variability (1.5%). The use of TBW, expressed as per kilogram (or using allometry with an exponent of 1), contributed only 32% of central volume of distribution between subject variability, but 82% on V2 between subject variability.

**Figure 5:**
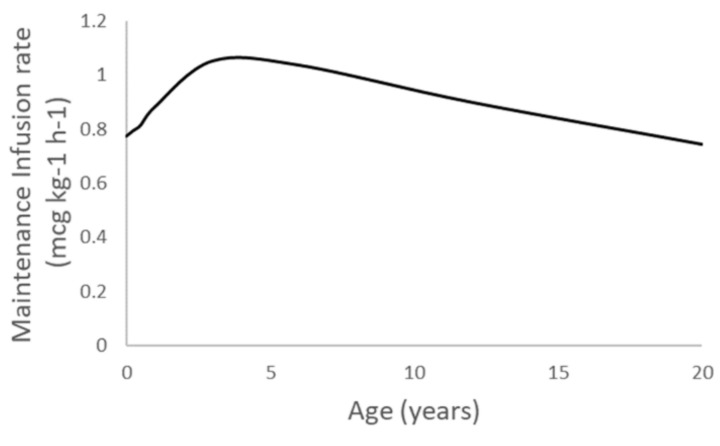
Simulated per kilo (TBW) dexmedetomidine maintenance infusion rates to maintain plasma concentration of 1 mcg/L. Infusion rates are affected by the effects of maturation and size in metabolic clearance. Clearance in term neonates is 42% of adult values, reaching 80% by 3 years of age. Allometric relationships between size and clearance explain the decrease in infusion rates (mcg/kg/min) in patients older than 3 years.

Dexmedetomidine maintenance infusion rates that maintain a simulated plasma concentration of 1 mcg/L are shown in Figure 5 and Table 3.

Infusion rates are affected by the effects of maturation and size on metabolic clearance. Clearance in term neonates is 42% of adult values, reaching 80% by 3 years of age. Allometric relationships between size and clearance explain the decrease in infusion rates (mcg/kg/min) in patients older than 3 years.

## 4. Discussion

Size and age were the two main covariates to describe variability in the dexmedetomidine data. We have derived a universal population PK model for dexmedetomidine that is applicable to both children and adults with a wide range of weights. We have shown that an adult model describing dexmedetomidine PK is adequate for use in children out of infancy, justifying the use of allometric theory. Size changes between individuals were explained by normal growth and the impact of obesity. The influence of size on dexmedetomidine PK was investigated with different scalars (TBW, FFM, NFM) using allometry. FFM is a measure that closely approximates lean body weight (LBW). FFM was a better size descriptor than TBW for clearance, consistent with other reports that have demonstrated LBW to be a suitable size descriptor to scale doses in obese patients [17]. In addition NFM, a scalar that allows adding a fraction of fat mass to FFM, was the most suitable size descriptor to describe dexmedetomidine changes in volumes of distribution.

Dexmedetomidine is metabolised in the liver by UGT1A4 and UGT2B10, aliphatic hydroxylation (CYP 2A6), and N–methylation (CYP2D6) [41]. There appears to be no evidence that dexmedetomidine interferes with the clearance of other medications that are substrates for CYP2D6 metabolism [42]. Dexmedetomidine is 93% protein-bound in children [24,41]. Most clinical PK studies of dexmedetomidine have been modelled using a two-compartment model. The rich data obtained in development of the Hannivoort model [7] made best use of a three-compartment model. Dexmedetomidine PK using pooled data were also better described with a three-compartment disposition model.

The clearance estimate of 0.9 (90% CI 0.81, 1.02) L/min/70kg using FFM as a scaler (56.1 kg, expected for a male with a TBM of 70 kg and height of 1.76 m) was consistent with that from Hannivoort for adults (41.16 L/h/70kg) [7] and Potts for children (42.1 95% CI 38.7, 45.8 L/h/70 kg; 0.7 L/min/70kg) [19]. The central compartment volume (V1) estimated in the Hannivoort study in healthy adults was small (1.78 L/70 kg), possibly due to the target concentration administration method and early blood sampling for assay [7]. Dexmedetomidine PK has been studied in adults, with similar three-compartment structural models yielding central volumes larger than 8 L [43,44,45,46,47]. A pooled study [19] in children obtained a V1 estimate of 56.3 L/70 kg, consistent with similar studies conducted in neonates, infants, and children [19,48,49,50,51]. Some of these data were sourced from children in intensive care, but estimates were similar to children out of intensive care and to adult intensive care patients. The central volume estimate for the current pooled analysis of 25.2 L/70 kg (CV% 104%; 95% CI 20.9, 31.3) is a more realistic value for a diverse population of patients than that reported by Hannivoort of 1.78 L/70 kg for healthy adults.

Haemodynamic changes induced by dexmedetomidine can produce peripheral vasoconstriction and reflex bradycardia, with a consequent reduction of V1. Dexmedetomidine produces a biphasic haemodynamic response [18], with hypotension at low plasma concentrations and hypertension at higher plasma concentrations. Hypertension predominates at the concentrations above 2.0 ng/ml that were frequently observed in the Hannivoort study. While vasoconstriction may contribute to the small V1 observed in the aforementioned study [7], a more important consideration is the haemodynamic instability induced by rapid infusion. It is for this reason that loading dose was estimated using simulated infusion over 30 min. Clearance is the main determinant of infusion rate. Consequently, infusion rates are affected by maturation and size. Clearance in term neonates is 42% of adult values, reaching 80% by 3 years of age. Allometric relationships between size and clearance explain the decrease in infusion rates (mcg/kg/min) in patients older than 3 years. Most enzyme systems responsible for metabolic dexmedetomidine clearance (e.g., glucuronidation (UGT 2B10, UGT1A4)) and by the cytochrome P450 (CYP 2A6) system [52]) are immature at birth and mature within the first few years of life. Consequently, drug CL is reduced at birth, but by the age of 1–2 years, it is greater than that observed in older children and adolescents (when expressed as per kilogram per unit of time).

We investigated the effect of body composition on dexmedetomidine PK using the distinction between fat and FFM. Measurements of FFM were only available from the Rolle [17] and Cortinez [28] data; FFM was predicted for all other individuals. There is unaccounted variability associated with these predictions, often attributed to the population characteristics and reference FFM measure by which the predictive formulae are derived. Estimates based on those proposed by Al-Sallami [33] are based on a population of children 3–11 years old and have not been validated in children under 3 years, an inconsistency also currently common to remifentanil universal models.

## Figures and Tables

**Table 1:** Dexmedetomidine population pharmacokinetic parameter estimates for the final universal model. Parameter estimates and population variability displayed as medians determined from 1000 bootstrap estimates.

Parameter	Estimate	PPV (%)	95% CI	Sh%
**V1 (L/70 kg)**	25.2	103.9	20.9, 31.3	16.4
**V2 (L/70kg)**	34.4	41.8	24.3, 44.2	15.5
**V3 (L/70 kg)**	65.4	61.6	53.4, 74.5	8.4
**CL (L/min/70 kg)**	0.897	35.8	0.81, 1.02	4.1
**Q2 (L/min/70kg)**	1.68	63.2	1.22, 1.97	12.5
**Q3 (L/min/70 kg)**	0.62	89.7	0.45, 0.83	21.4
**FFATV**	0.293	-	0.13, 0.55	-
**FFATCL**	0 FIX	-	-	-
**TM_50_**	52.4	-	43.5, 68.8	-
**Hill**	1 FIX	-	-	-
**Additive Residual Error (µg/mL)**	0.004	η_RUV_ 0.32	-	
**Proportional Residual Error (%)**	0.19	-	0.18, 0.20	

Volume of distribution: V; clearance: CL; intercompartmental clearance: Q; TM_50_: maturation halftime; Hill: exponent describing the steepness of the maturation profile. FFATV: factor on fat for volume; FFATCL: factor on fat for clearance. Residual unidentified variability: RUV; population parameter variability: PPV%. Sh% = shrinkage. Size is accounted for using theory-based allometric scaling to a 70 kg individual with the allometric exponents of ¾ for CL and 1 for V. PPV% = √variance.

**Table 2:** Effect of covariate analysis on variance (*ω*^2^). Impact of each covariate on CL when added sequentially to the model.

Sequential Nested Model	PPVt^2^	BSVR^2^	BSVP^2^	BSVP^2^/PPVt^2^
Clearance				
no covariates	0.861 *	0.861 *	0	0
TBW with allometric scaling (EXP = 3/4)	0.861 *	0.140	0.721	0.838
TBW with PMA on CL	0.861 *	0.136	0.725	0.842
FFM with PMA on CL	0.861 *	0.114	0.747	0.867
Central compartment (V1)				
no covariates	1.5 *	1.5*	0	0
TBW allometric scaling (EXP = 1)	1.5 *	1.02	0.48	0.320
Peripheral compartment (V2)				
no covariates	1.46 *	1.46*	0	0
TBW allometric scaling (EXP = 1)	1.46 *	0.25	1.209	0.823

* = assumed from no covariate model estimate. PPVt is the total population variability; BSVR is the random BSV estimated on a parameter when covariate analysis is included; BSVP is the population between subject variability; TBW is total body weight; PMA is postmenstrual age; FFM is fat free mass.

**Table 3:** Dexmedetomidine maintenance infusion rates, determined using simulation, that maintain plasma concentration of 1 mcg/L. The loading dose was given over 30 min and the maintenance infusion scheme was designed to maintain dexmedetomidine a plasma concentration of 1 mcg/L.

Age	Weight (kg)	Height (cm)	Clearance (L/min)	Loading Dose (mcg/kg)	Maintenance (mcg/kg/h)
Term neonate	3.6	50	0.05	0.40	0.77
3 months	6	62	0.08	0.38	0.80
6 months	7.8	67	0.11	0.37	0.81
1 year	10	75	0.15	0.37	0.88
3 years	14	95	0.25	0.40	1.04
6 years	21	115	0.36	0.39	1.02
12 years	40	149	0.60	0.35	0.90
20 years	70	175	0.87	0.31	0.75

## Supplementary Materials

The following are available online at https://www.mdpi.com/2077-0383/9/11/3480/s1, Table S1: Study characteristics from the pooled paediatric data used to develop the original dexmedetomidine Potts PK model. Data represent count or mean (range). Table adapted from Potts [19]. Figure S1: Time profiles of the observed/predicted ratios of dexmedetomidine plasma concentrations determined using the model by Hannivoort et al. [7]. Observed data were sourced from Potts 2009 [19]. Each blue line represents a single participant. The broken grey lines demonstrate the acceptable range of dexmedetomidine observed/predicted ratios. The solid black line represents optimal predictive performance of the model (i.e., identical observed and predicted values). The red line demonstrates the median observed/predicted ratio and was determined with a loess smoothing function. Figure S2: Maturation of dexmedetomidine clearance when scaled using total body weight.
