# Supplementary material for: A Universal Pharmacokinetic Model for Dexmedetomidine in Children and Adults
Source: J Clin Med. 2020 Oct 28;9(11):3480. doi: 10.3390/jcm9113480 (PMC7692360; doi:10.3390/jcm9113480)
Supplement: Supplementary file 1 [file jcm-09-03480-s001.pdf]

Table S1. Study characteristics from the pooled pediatric data used to develop the original dexmedetomidine Potts PK model. Data represent count or mean (range). Table adapted from Potts [20].

Study	*n*	Age (y)	Weight (kg)	Procedure	Route of Administration	Dose	Sampling schedule	Source
**1**	24	5.6 (2.0–12.0)	16.8 (10–42)	Urologic, lower abdominal or plastic surgery	Slow bolus over 10 min	2, 4 or 6 μg/kg	10, 15, 30, 75 min, 2.5, 4, 6, 12, 24 h	[25]
**2**	16	2.75 (0.2–10.0)	14.58 (5.3–37.5)	Bronschoscopy or NMRI	Slow bolus over 5 min	1 μg/kg	5, 10, 20 min, 1, 2, 3, 4, 5 h	[26]
**3**	45	3.38 (0.01–14.4)	15.1 (3.1–58.9)	Cardiac surgery	Slow bolus over 10 min	1–4 μg/kg	5–30 min, 1–2, 3–4, 6–10 h	[17]

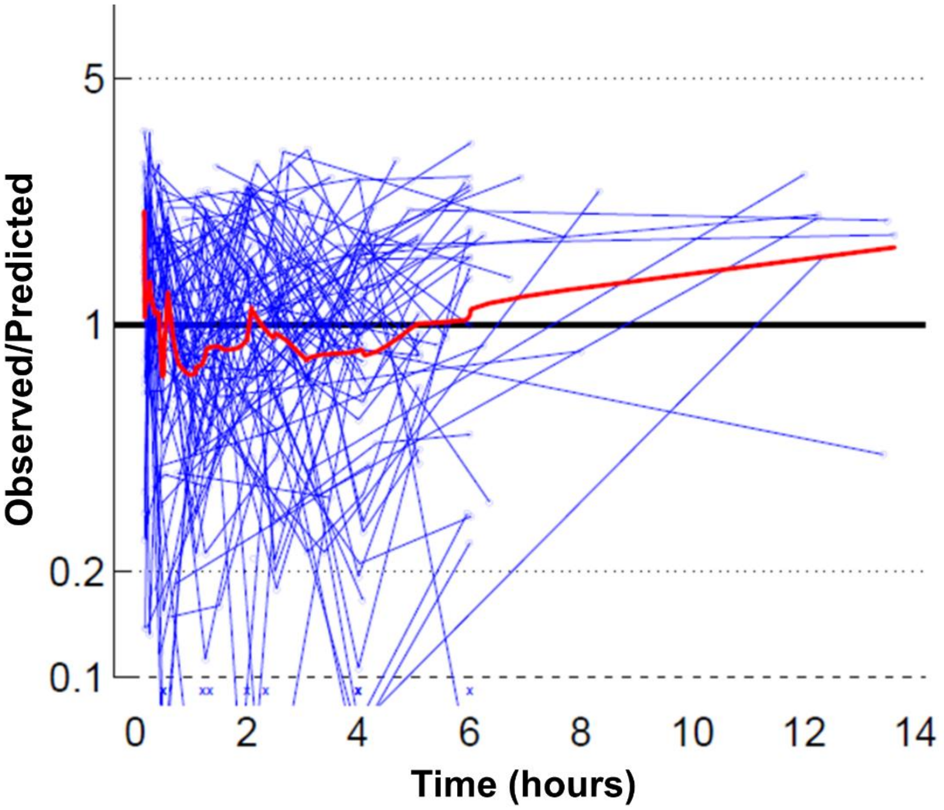

Figure S1. Profiles of the observed/predicted ratios of dexmedetomidine plasma concentrations determined using the model by Hannivoort et al.[8] Observed data were sourced from Potts 2009.[20] Each blue line represents a single participant. The broken grey lines demonstrate the acceptable range of dexmedetomidine observed/predicted ratios. The solid black line represents optimal predictive performance of the model (i.e. identical observed and predicted values). The red line demonstrates the median observed/predicted ratio and was determined with a loess smoothing function.

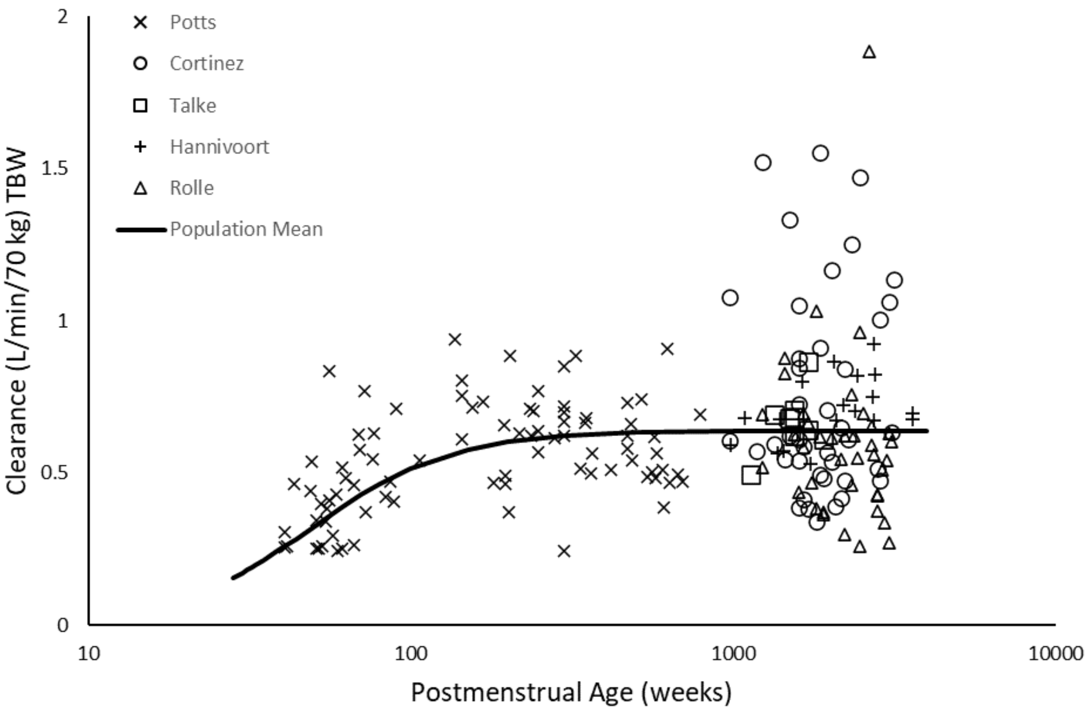

Figure S2. Maturation of dexmedetomidine clearance when scaled using total body weight.
